# Couplet medicines of leech and centipede granules improve erectile dysfunction via inactivation of the CaSR/PLC/PKC signaling in streptozotocin-induced diabetic rats

**DOI:** 10.1042/BSR20193845

**Published:** 2020-02-04

**Authors:** Jian Xiong Ma, Bin Wang, Cai Fei Ding, Hai Song Li, Xue Juan Jiang, Chen Ye Wang, Jia Yu, Wang Qiang Chen

**Affiliations:** 1 Department of Reproductive Medicine, Zhejiang Integrated Traditional and Western Medicine Hospital, Zhejiang, China; 2 Department of Andrology, Beijing University of Chinese Medicine Affiliated Dongzhimen Hospital, Beijing, China

**Keywords:** apoptosis, CaSR/PLC/PKC signaling pathway, diabetes mellitus, endothelial cells, erectile dysfunction

## Abstract

Erectile dysfunction (ED) is one of the significant complications of diabetes mellitus (DM), and CASR plays an important role in cellular antiapoptosis and NO production in the vascular endothelium by activating PKC. The present study was aimed to investigate the efficacy of Leech and Centipede Granules (LCG) through the CaSR/PLC/PKC signaling. Fifty male Sprague-Dawley rats were treated with streptozotocin to induce the DM model. After 10 weeks, an apomorphine test was used to confirm DMED. Rats with DMED were administrated with LCG and U73122 for 4 weeks. Fasting blood glucose, body weight, insulin and glucagon levels were measured. Erectile function in rats was assessed by apomorphine. Serums were measured using enzyme-linked immunosorbent assay and flow cytometry, and penile tissues were harvested for histologic and the expression of related targets analyses. After treatment, fasting blood glucose, body weight, insulin, glucagon levels, and erectile function were significantly ameliorated in the LCG groups. The LOX-1, NOX, and EMPs concentrations were significantly decreased with LCG treatment. LCG also continuously increased NO and decreased ET-1 content in penile tissues. LCG and U73122 administration also improved penile fibrosis by significantly decreasing VCAM-1, ICAM-1, and CD62P. The data also showed that LCG reduced the apoptosis level in the penis. Furthermore, the inhibited activation of the CaSR/PLC/PKC pathway was observed in DMED rats with LCG treatment. Collectively, LCG significantly ameliorated erectile function of DMED rats via increased NO generation, inhibiting endothelial cells apoptosis and penile fibrosis, which might benefit from the suppression of CaSR/PLC/PKC pathway in DMED rats.

## Introduction

Erectile dysfunction (ED) is characterized by the distinctive inability to achieve or maintain a sufficient erectile function for satisfaction during sexual activity [1], which was also accompanied by hypertension, coronary atherosclerosis, and diabetes mellitus [2]. ED is also known as impotency in men, estimated on 322 million men would suffer from ED global by the year 2025 [3], threatening human health, spousal relationship, and family life. Among diseases, diabetes mellitus (DM) is a chronic metabolic disease characterized by hyperglycemia, and which is showed to be significantly associated with ED. It has been noted that the incidence of ED in diabetic patients has risen nearly 3-fold when compared with non-diabetic men [2,4]. In addition, approximately 50% of men with diabetes noted ED within 10 years of diagnose [5,6]. The pathophysiology of diabetes mellitus-induced erectile dysfunction (DMED) is multifactorial and not fully enucleated. Obviously, it is believed that a high glucose environment caused damage to the central nervous system plays a crucial role in the development of DMED [7]. In brief, hypogonadism, advanced glycation end product (AGE) formation, vascular endothelial dysfunction, and decreased production of nitric oxide (NO) [8] are considered the main functional impairments in cavernoursal tissues. Cavernous endothelium involved in the cavernosal smooth muscle cell relaxation, which is a basic physiological phenomena contributes to the initiation and maintenance of erectile function [9]. Moreover, endothelial dysfunction and fibrosis were identified as a common pathogenic factor under high glucose levels in DMED [9–11]. Unfortunately, the exact mechanism of this effect of DMED still remains a mystery.

Protein kinase C (PKC) is serine/threonine kinase that belongs to a protein family and consisted of three subfamilies: conventional PKCs, novel PKCs, and atypical PKCs. Several studies reported that PKC activation can strengthen the NADPH-oxidases (NOX) activity, and further promote reactive oxygen species (ROS) production [12]. Studies have demonstrated that PKCδ, one of the novel PKCs activated by diacylglycerol or 12-O-tetradecanoylphorbol 13-acetate under oxidative stress, whose activation is associated with various biological events, including endothelial cells dysfunction, cell proliferation, and apoptosis, and vascular smooth muscle cells contractility [13]. As the study of Durpes et al. [14] demonstrated that PKC activation promotes endothelial cells dysfunction by inhibiting IL-18/IL-18BP pathway for exacerbated the progress of atherosclerotic in diabetes. Additionally, over-generation of ROS can lead to diminished NO bioavailability [15]. NO is the primary neurotransmitter of corporal smooth muscle relaxation, which plays a significant role in penile erection [2]. A unifying opinion suggests that the initiation and development of DMED may be involved in impaired NO synthesis [16]. Besides, NO is synthesized by endothelial NOS (eNOS), inducible NOS (iNOS), and nNOS. Additionally, the iNOS contributes to the synthesis NO incessantly depend on Ca^2+^ [17]. The Ca^2+^ concentration was detected in the blood by using the calcium-sensing receptor (CASR), which is a class C G-protein coupled receptor (GPCR), and typified by 1078-amino acid as a disulphide-linked homodimer [18]. Recent evidence indicates that CASR combined with multiple G-protein subtypes for activating predominantly signals. For instance, Conigrave [19] and Brennan et al. [20] reported that the CaSR coupled to the Gq/11-phospholipase C (PLC)-mediated pathway, to generate inositol 1,4,5-trisphosphate (IP3) and diacylglycerol (DAG) for activating PKC and Ca^2+^ mobilization as well as mitogen-activated protein kinase (MAPK) cascades including JNK and p38. Further, the JNK and p38 also mediated apoptosis that accompanied by increased Bax levels in the vascular endothelium cells [21]. Therefore, the CaSR/PLC/PKC signaling pathway may play a crucial role in the erectile dysfunction associated with streptozotocin-induced diabetic rats.

Currently, phosphodiesterase type 5 (PDE5) inhibitors that help augment the NO/cyclic guanosine monophosphate (cGMP) pathway are used as the first-line therapeutic strategy for ED. However, studies have reported that these drugs are not effective in some diabetic male patients with severe ED [22] and result in several side effects, including facial redness, headache, and gastrointestinal reactions [4]. Thus, exploring novel therapeutic strategies and targets for DMED is still urgent. ED is classified as “Yangshuo” syndrome in traditional Chinese medicine (TCM), and TCM scholars believe that the “qi stagnation and blood stasis” is the main pathological features of DMED. Previously, we reported that the “Huoxue Tongluo Qiwei soup” granules (HTQGs) was achieved a significant therapeutic efficiency of 76% in clinical trials [23], and composed of *Leech* (10 g), *Centipede* (5 g), *Radix Paeoniae Rubra* (20 g), *Vaccariae Semen* (10 g), *Angelicae Sinensis Radix* (15 g), *Radix Bupleuri* (15 g), *Cyathulae Radix* (15 g), *Tribulifructus* (20 g), *Citri Reticulatae Pericarpium Viride* (10 g), *Curcumae Radix* (10 g), *Morindae Officinalis Radix* (15 g), and *Epimrdii Herba* (10 g). Among them, the couplet medicines of *Leech* and *Centipede* is the key medicines for the purpose of promoting blood circulation and removing blood stasis in TCM, and also the core herbs in HTQs.

Given the beneficial effects of couplet medicines of Leech and Centipede in the treatment of DMED and rare studies correlating the CaSR/PLC/PKC signaling axis with DMED, the present study aims to establish DMED rat models to investigate the effect of Leech and Centipede Granules (LCG) and the possible underlying mechanism of DMED in penis tissue, which might be a beneficial treatment strategy of DMED in human.

## Materials and methods

### Experimental animals

Fifty-six 12-week-old male Sprague-Dawley (SD) rats (weight, 260–280 g) were purchased from Shanghai SLAC Laboratory Animal Co.,Ltd (Shanghai, China). All rats were raised in the Animal Center of Zhejiang Chinese medical university (Zhejiang, China) with a 12/12 light-dark cycle at 24°C ± 1°C, water and food available ad libitum. The current experimental protocols were approved by the Animal Care and Use Committee of Zhejiang Chinese medical university (Zhejiang, China). The mating test was conducted and showed that all rats possessed the normal erectile function. Diabetes was induced by sustaining a high-fat diet (HFD) feeding routine for a month. Then, after an overnight fast, 50 SD rats were injected with a single intraperitoneal injection of 60 mg/kg streptozotocin (STZ, Sigma-Aldrich Chemical Co, St. Louis, MO, U.S.A.). Six age-matched rats only got an intraperitoneal injection of 0.1 mol/l citrate–phosphate buffer (pH 4.5) and selected as a control group. Rats with a constant non-fasted blood glucose concentration ≥16.7 mmol/l were considered diabetic after 72 h. The diabetic rats were fed for 10 weeks to develop ED. Next, apomorphine (APO)-induced erection test was performed to evaluate the erectile function. The rats were moved to a quiet and dimly laboratory to adapt to the environment for 15 min in a transparent observation kit. Then, the rat soft skin of the neck region was injected with a one-off injection with 100 μg/kg of APO (Shenyang, Liaoning, China). The status and frequency of penile erection in rats were observed for 30 min, and the penis was enlarged, the prepuce was receded or the glans was exposed represented one erection [24]. Rats with abnormal erectile function were defined as having DMED.

Finally, 36 DMED rats were identified for the subsequent experiments. The DMED rats were divided randomly into six treatment groups (*n* = 6): the DMED model group, low-dose group, middle-dose group, high-dose group, HTQG group, and the phospholipase C (PLC) inhibitor U73122 group. The low-, middle- and high-dose group rats have received a daily gavage of LCG (Huisong Pharmaceuticals Co., Ltd, Zhejiang, China) at a dose of 0.35 g/kg, 0.7 g/kg, and 1.4 g/kg for 4 weeks, respectively. Besides, the HTQG group rats were administered daily with the prescription of “Huoxue Tongluo Qiwei soup” granules at a dose of 3 g/kg. For the U73122 group rats, there was 10 mg/kg PLC inhibitor was injected in the tail vein of rats every one day for 4 weeks. The control and DMED model group received physiological saline only. At the end of the study, all rats were fasted for 10 h, then the tail vein blood glucose levels, body weights and erectile function of all rats were measured. After that, all rats were anesthetized with pentobarbital sodium (50 mg/kg, i.p. Sigma), then blood sample was collected from the abdominal aorta and centrifuged at 3000 rpm/min for 15 min to acquire the sera. Subsequently, the rats were killed by decapitation, then the penile tissues were harvested stored at −80°C until further analysis.

### Erectile function assessment

Four weeks after treatment, rats in each group were placed in a transparent observation cage with dimmed lights and a quiet environment that allowed observation. Then, the rats were injected with 100 μg/kg APO in the neck. The number of penile erections in rats was observed and recorded for 30 min.

### Measurement of insulin, glucagon, lectin-like oxidized low-density lipoprotein receptor-1, NADPH oxidase, NO, and endogenous ET-1

Levels of insulin, glucagon, lectin-like oxidized low-density lipoprotein receptor-1 (LOX-1), and NADPH oxidase (NOX) in rats sera were measured using commercial insulin, glucagon, LOX-1, and NOX ELISA Kit, respectively (Solarbio, Beijing, China). Furthermore, NO and ET-1 concentrations in penile tissues were estimated with a total NO assay kit (Beyotime, China) and ET-1 Elisa kit (Jianglai Biotechnology Co., Ltd., Shanghai, China) according to the manufacturer’s procedure.

### Flow cytometry

Blood samples were collected from all rats in each group by clean arteriopuncture into 2 ml vacutainer tubes under strict sterile conditions. The samples were centrifuged a 1000 rpm for 20 min to obtain platelet-rich plasma (PRP). Then, the PRP was centrifuged at 12000 rpm for 5 min to prepare platelet-poor plasma (PPP). Phenylethylamine (PE) anti-Rat CD31-PECy7 (eBioscience, U.S.A.) and allophycocyanin (APC) labeled anti-mouse/rat CD42b (Biolegend, Germany) were used to detect endothelial microparticles (EMPs). About 50 μl PPP were incubated with 8 ml of each labeled antibodies for 30 min at 4°C in the dark. The stained samples were resuspended in 0.2 ml of PBS and then kept for flow cytometry analysis.

For flow cytometric detection of vascular cell adhesion molecule (VCAM)-1, intracellular adhesion molecule (ICAM)-1, and selectin P (CD62P) in cavernous tissue. The cavernous tissue was digested to prepare 1 × 10^6^/ml single-cell suspension. Cells were incubated for 30 min in PBS containing anti-rat CD106-PE (VCAM-1), anti-rat CD54-PE (ICAM-1), and anti-mouse/rat CD62P-APC (P-selectin). The stained cells were then detected by the MACSQUANT Q10 cytometer (Miltenyi Biotec) and analyzed using FlowJo v7.6.5 software (Tree Star).

### Hematoxylin–eosin (HE) and immunohistochemical staining

Freshly dissected central parts of penile tissues were fixed in 4% paraformaldehyde. The sample tissues were then dehydrated by an ethanol gradient and embedded in paraffin. About 4 µm thick sections were prepared for HE staining as well as immunochemistry (IHC) according to standard protocols for histological examinations. Sections were cut at 4 μm and incubated at room temperature for 4 h with primary antibodies against Bax (1:250, Abcam), Bcl-2 (1:500, Abcam), Caspase-3 (1:500, Abcam) and eNOS (1:100, Abcam). Next, the sections were washed and incubated with appropriate secondary antibodies for a 15 min incubation. Afterward, the sections were incubated with 3,3-diaminobenzidine (DAB), and the cell nuclei were stained with hematoxylin. Finally, sections were examined under a light microscope. The semi-quantitative analysis of the expression of Bax, Bcl-2, Caspase-3, and eNOS in penile tissue in the images was measured by using Image-Pro Plus version 6.0 software (Media Cybernetics Inc., MD, U.S.A.).

### Real-time PCR

The total RNA from rat penile tissues was extracted using Trizol reagent (Life Technologies, Grand Island, NY, U.S.A.) according to the manufacturer’s protocol. The cDNA was synthesized utilizing the reverse transcription kit (Fermentas, Waltham, MA, U.S.A.). qRT-PCR was performed using a LightCycler® 96 Real-Time PCR System (Roche, Switzerland) with the SYBR Green PCR kit (Takara, Japan). The program was comprised of denaturation at 95°C for 10 min, trailed by 40 cycles of liquefying at 95°C for 15 s and prolongation at 60°C for the 60 s. Primer sequences are shown in Table 1. The level of GAPDH mRNA expression was measured as an endogenous reference and used for normalization. The relative quantification of the expression levels of the target genes was calculated using the 2^−ΔΔCt^ method.

**Table 1:** The primer sequences for qRT-PCR

Gene	Forward Primer (5′‐3′)	Reverse Primer (5′‐3′)
JNK	GCCGGAGGTGATTTTGGGTA	GTAACGGGGCGATAACGGAT
P38	CGGTGTGTGCTGCTTTTGAT	CAGACGCAACTCTCGGTAGG
BAX	GAGCTGCAGAGGATGATTGCT	TGATCAGCTCGGGCACTTTA
CASR	CACCCAAGTGGAGAAAACGC	CTGAACTATTGGCAACGCCG
PLC	CCCACCACCCAGGAAATTGT	GGTTTTGCTTGCTCAGGTGG
PKCδ	GAAAGCCACACTGAATCCCG	ACTTGTACCATCCATCCACAGG
GADPH	GCGGGAGCGGATCCTAATA	TGGTGCATCCATGGGCTAC

JNK: c-Jun N-terminal kinase JNK; MAPK14 (Also known as P38): mitogen-activated protein kinase 14; BAX: BCL2-associated X protein; CASR: Calcium-sensing receptor; PLC: phospholipase C; PKCδ: Protein kinase C δ; GADPH: glyceraldehyde-3-phosphate dehydrogenase; qRT‐PCR, quantitative real‐time polymerase chain reaction.

### Western blotting

Total protein samples were extracted from penile tissues. The penile tissues were homogenized by precooled tissue lysates and centrifuged at 12,000 rpm for 15 min at 4°C, and the supernatants were obtained. Protein concentrations were determined using a BCA protein assay kit (Solarbio, Beijing, China). Protein lysate of equal concentration was loaded on 10% sodium dodecyl sulfate/polyacrylamide gel electrophoresis (SDS-PAGE) at 80 V for 30 min and 120 V for 60 min, and then electrotransferred to a polyvinylidene difluoride membrane (Bio-Rad, CA, U.S.A.). The membrane was blocked for 1 h at room temperature with 5% skimmed milk powder. Then, membranes were incubated with primary antibodies against CaSR (1:1000; ab18200, Abcam), PLC (1:5000; ab76155, Abcam), PKCδ (1:5000; ab182126, Abcam), JNK (1:2000; ab208035, Abcam), p38 (1:1000, ab170099, Abcam), Bax (1:2000, ab32503, Abcam), GAPDH (1:5000, 60004-1-1g, HuaBio, Hangzhou, China) at 4°C overnight, followed the membranes were washed and incubated with the appropriate horseradish peroxidase-conjugated secondary antibodies at room temperature for 1 h. Finally, the protein bands were visualized using an enhanced chemiluminescence detection system (Clinx Science Instruments, U.S.A.).

### Statistical analysis

All data are expressed as mean ± standard deviation. Groups were compared using Student’s *t*-test or one-way ANOVA. All data were analyzed using SPSS 22.0 statistical software (SPSS Inc., Chicago, IL, U.S.A.) and *P* < 0.05 was considered to be statistically significant.

## Results

### LCG improves the erectile functioning of rats with DMED

**Figure 1:**
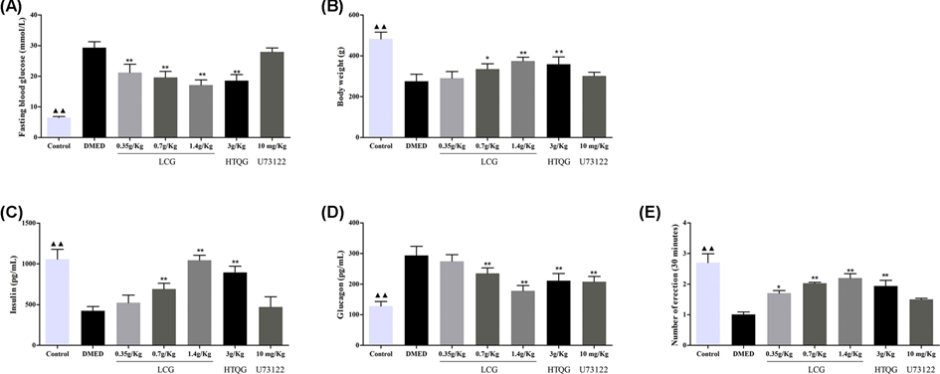
Comparisons of fasting blood glucose, body weight, insulin, glucagon, and the times of erection in rats with DMED (**A**) The fasting blood glucose level is decreased in LCG and HTQG groups. (**B**) The body weight is increased in LCG and HTQG groups. (**C**) The insulin levels of rats are expressed at a high level with LCG treatment. (**D**) The glucagon level is expressed at a low level in treated groups, compared with the DMED group. (**E**) APO experiments are performed to evaluate erectile function in each group. Data are expressed as mean ± standard deviation from *N* = 6 per group. ^▲▲^*P* < 0.01 the Control group versus the DMED group; *P < 0.05, **P < 0.01 the LCG, HTQG, and U73122 groups versus the DMED group.

Fasting blood glucose level and body weight changes are shown in Figure 1. After diabetes induction, the fasting blood glucose level was found to be significantly elevated of rats in the DMED group (*P* < 0.01, Figure 1A) but significantly lower body weights (*P* < 0.01, Figure 1B). LCG treatment improves these differences. In comparison with the DMED group, the insulin levels of in rats in the LCG groups was found to dramatically increased after LCG administration (*P* < 0.01, Figure 1C), whereas the glucagon levels of rats in LCG, HTQG, and U73122 groups were decreased (*P* < 0.01, Figure 1D). The result of APO-induced erection testing is shown in Figure 1E. The number of erection for 30 min of rats in LCG and HTQG groups was found to be significantly increased with a dosage dependence. Compared with the DMED group, there were no significant differences in the fasting blood glucose level, body weight, insulin levels, and the times of erection in the U73122 group. These findings suggested that the LCG has a defensive effect on ED in the diabetic penis.

### LCG inhibits endothelial cells injury and ameliorates vascular endothelial relaxation of rats with DMED

**Figure 2:**
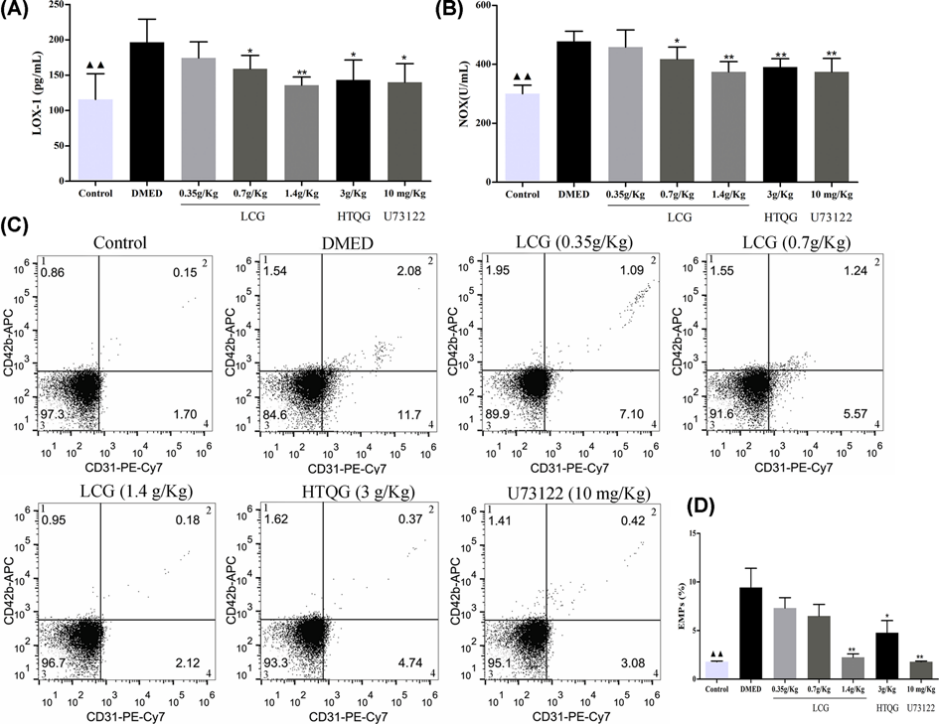
Effect of LCG on endothelial cells injury of rats with DMED (**A,B**) Evaluation of LOX-1 and NOX concentration in DMED rats sera by Elisa. (**C**), four-quadrant histogram to detect markers with negative for CD42b and positive for CD31 in quadrant 4. (**D**) Quantification of CD42b- CD31+ EMPs by flow cytometry in DMED rats sera. Measurement data were expressed as mean ± SD, ^▲▲^*P* < 0.01 the Control group versis the DMED group; *P < 0.05, **P < 0.01 the LCG, HTQG, and U73122 groups versus the DMED group.

**Figure 3:**
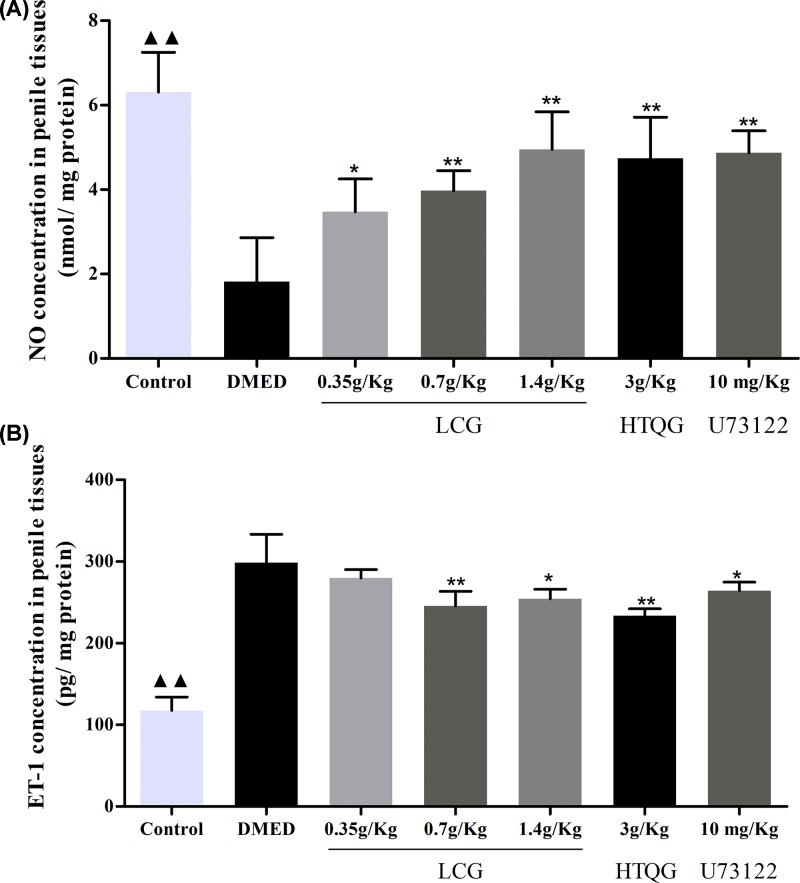
Effect of LCG on vascular endothelial relaxation of penile tissue in rats with DMED (**A**) The NO concentration and (**B**) ET-1 of penile tissue measured by Elisa, and represented by mean ± standard deviation; the one-way ANOVA was performed to analyze data in each group. ^▲▲^*P* < 0.01 the Control group versus the DMED group; *P < 0.05, **P < 0.01 the LCG, HTQG, and U73122 groups versus the DMED group.

Some reports indicate that the LOX-1 and NOX are the important marker evaluation of vascular endothelial cell injury. In the present study, results of LOX-1 (Figure 2A) and NOX (Figure 2B) detection displayed that compared with the control group, the LOX-1 and NOX concentrations were markedly elevated in the DMED group (all *P* < 0.01), while it was partly reduced in the middle-dose group, high-dose group, HTQG group, and the U73122 group. Besides, there were no significant differences in the levels of the low-dose group compared with the DMED group. EMPs, the cell plasma fragments, directly reflecting the physiological process of the endothelial cell, including cell activation, proliferation, and apoptosis [25]. In the present study, four quadrants histogram was used to differentiate EMPs, being negative for CD42b and positive for CD31. The flow cytometry analyses showed that the EMPs counts were significantly decreased in the 1.4 g/kg LCG, HTQG and U73222 groups compared with the DMED group (Figure 2C,D). Also, NO concentration in penile tissues of the LCG-treated group was significantly elevated compared with the DMED group (Figure 3A). Furthermore, the ET-1 concentration of penile tissues in treated rats was significantly lower than that in DMED group (*P* < 0.05*; P* < 0.01, Figure 3B). Taken together, the results show that LCG could inhibit the pathological process of endothelial cell impairments and ameliorate vascular endothelial relaxation of penile tissue in DMED.

### LCG reduced vascular fibrosis markers expression in the sera of DMED rats

**Figure 4:**
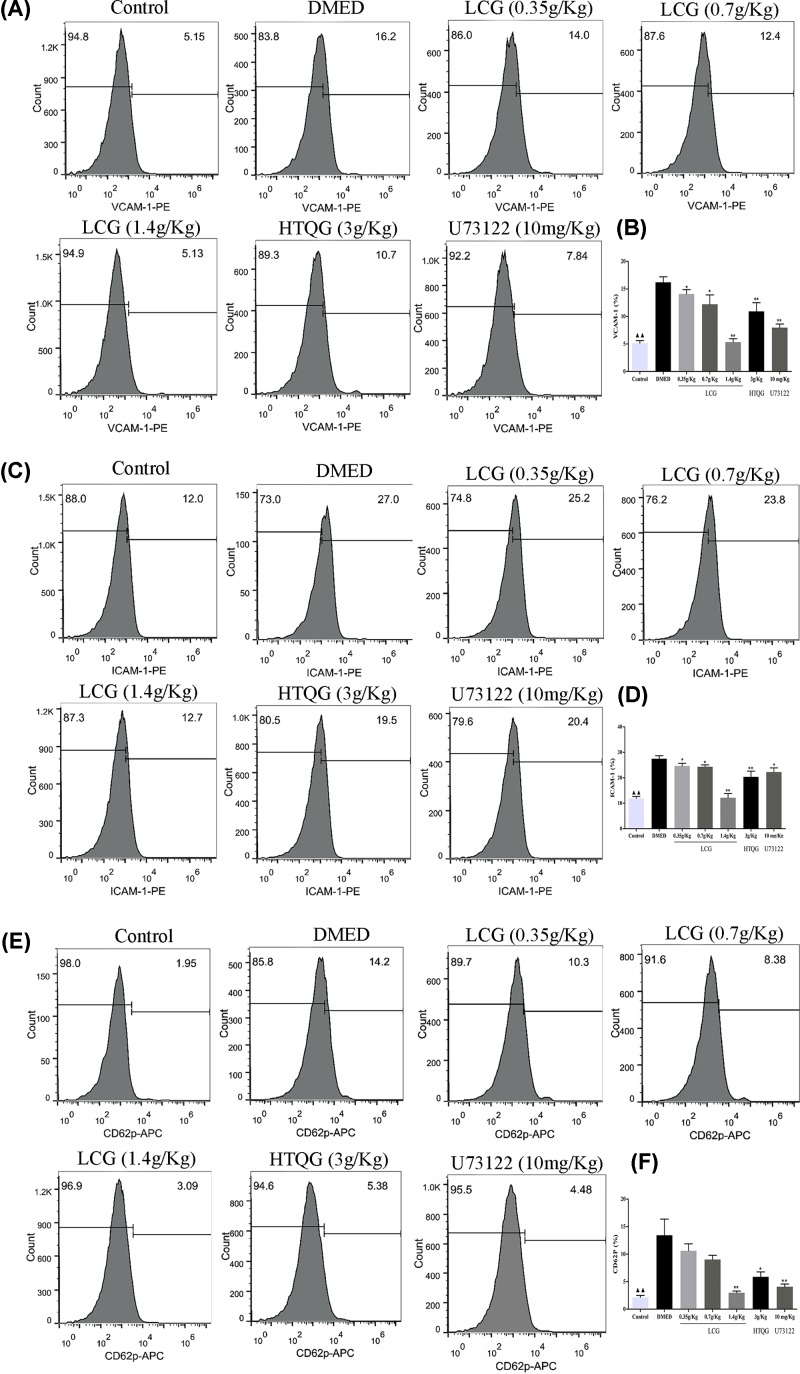
Effect of LCG on vascular fibrosis in rats with DMED (**A,C,E**) Flow cytometry measured the VCAM-1, ICAM-1, and CD62P level after LCG treatment for 4 weeks. (**B,D,F**) Bar graph demonstrated comparison of VCAM-1, ICAM-1, and CD62P concentration. Data presented as mean ± SD; the comparison of multiple groups was performed by ANOVA; ^▲▲^*P* < 0.01 the Control group versus the DMED group; *P < 0.05, **P < 0.01 the LCG, HTQG, and U73122 groups versus the DMED group.

Previous studies have revealed that proposed biomarkers, VCAM-1, ICAM-1, and CD62P with higher concentrations are independently associated with fibrosis [26,27]. To investigate the role of LCG on the vascular fibrosis in DMED rats, the VCAM-1, ICAM-1, and CD62P levels in serum samples of rats were assayed with flow cytometry. As shown, compared with the control group, the count of VCAM-1 (Figure 4A,B), ICAM-1 (Figure 4C,D), and CD62P (Figure 4E,F) were increased in the DMED group (*P* < 0.01). Intriguingly, flow cytometry analysis showed that the serum of rats in the LCG, HTQG, and U73122 group notably decreased levels of VCAM-1, ICAM-1, and CD62P in a dose-dependent manner compared with DMED rats group. These results suggest that LCG and HTQG were determined to have exerted a greater effect on the amelioration of vascular fibrosis in DMED rats.

### LCG alleviates the pathology of cavernous bodies

**Figure 5:**
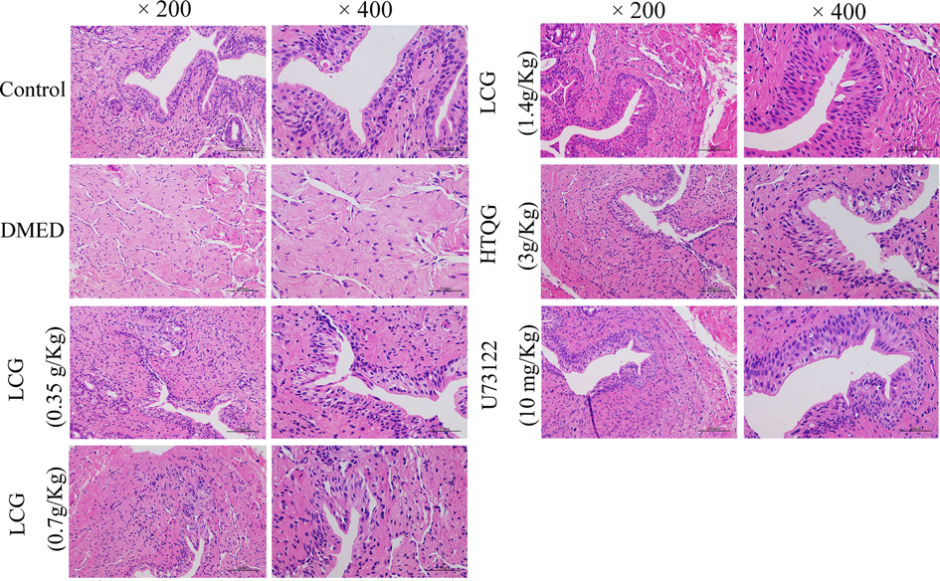
Effect of LCG on the pathological progression of DMED Pathological changes of cavernous bodies of rats treated with LCG at the dosage of 0.35 g/kg, 0.7 g/kg, and 1.4 g/kg were observed by hematoxylin and eosin staining (200× and 400×).

The present study assessed the effects of LCG and HTQG on the pathology of cavernous bodies. A large number of the cavernous sinus and endothelial cells were observed in the corpus cavernosum tissues of rats of control, LCG, HTQG, and U73122 groups but not in the DMED group (Figure 5). In comparison with the DMED group, the number of the cavernous sinus and endothelial cells in the cavernosum tissues of the treated group was observed to be significantly improved, while the rats of DMED group with obviously decreased numbers. In the concern of the above results, we can demonstrate that LCG and HTQG can improve the pathological changes of cavernous bodies of the penis by depressing the endothelial cell lesion.

### Immunohistochemical analysis of Bax, Bcl-2, Caspase-3, and eNOS expression

**Figure 6:**
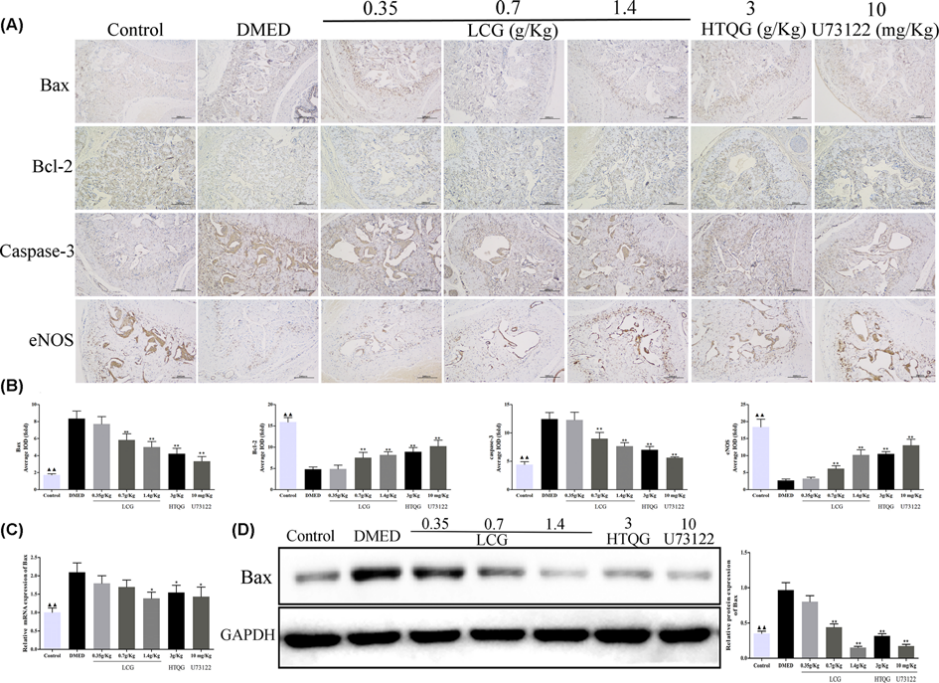
Evaluation of Bax, Bcl-2, Caspase-3, and eNOS protein expression by immunohistochemistry (**A**) Representative results of IHC staining for Bax, Bcl-2, Caspase-3, and eNOS in penile tissue (original magnification, 100×). (**B**) Representative statistical analysis of Bax, Bcl-2, Caspase-3, and eNOS in the penile tissue among the groups. (**C**) RT-qPCR results show that Bax was expressed at a high level in DMED rats, while decreased by LCG, HTQG, and U73122 treatment. (**D**) Protein bands of Bax, and GAPDH, and representative statistical analysis of Bax. Each bar depicts the mean values (mean ± s.d.); *^▲▲^P* < *0.01* the Control group versus the DMED group; **P < 0.01 the LCG, HTQG, and U73122 groups versus the DMED group.

The protein expression levels of Bax, Bcl-2, Caspase-3, and eNOS in rat penile tissue endothelial cells were estimated by immunohistochemistry. As shown in Figure 6A,B. Immunohistochemical analysis revealed that the protein expression of Bax and Caspase-3 were significantly higher in the rats from the DMED group compared with those from the control group (*P* < 0.01). However, the protein expression of Bcl-2 and eNOS was significantly lower than that in control rats. After treatment, the protein expression of Bax and Caspase-3 was significantly decreased in the treated group rats than that in the DMED group, and significantly increased the protein levels of Bcl-2 and eNOS in the treated groups with dose-dependence. Of note, the protein expression levels of Bax, Bcl-2, Caspase-3, and eNOS in the low-dose LCG group did not present with a statistical difference. Additionally, the results of RT-qPCR and Western blot analysis showed that the mRNA and protein expression of Bax was significantly decreased in the high-dose group, HTQG, and U73122 group (Figure 6C,D).

### LCG inhibited the CaSR/PLC/PKC signaling pathway and the expression of JNK, P38, and Bax are decreased in DMED

**Figure 7:**
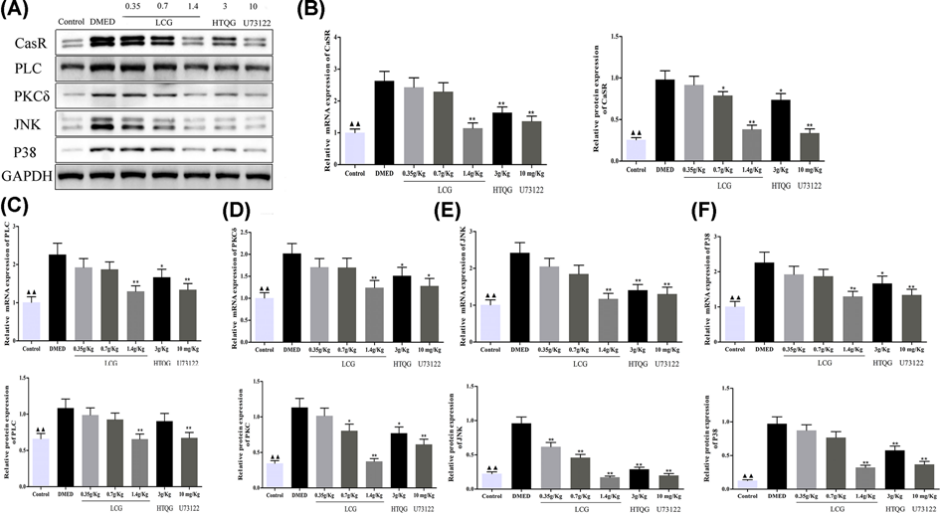
Expressions of CaSR, PLC, PKC, JNK, and P38 detected by RT‐qPCR and Western blot analysis (**A**) Protein bands of CaSR, PLC, PKC, JNK, P38, and GAPDH. (**B**–**F**) RT-qPCR and Western blot analysis of CaSR, PLC, PKC, JNK, and P38 were expressed at a high level in DMED rats, while reversed by LCG, HTQG, and U73122 treatment, respectively. Statistical data are presented as mean value ± SD. *^▲▲^P* < 0.01 the Control group versus the DMED group; **P < 0.01 the LCG, HTQG, and U73122 groups versus the DMED group.

To investigate the expressions of CaSR, PLC, PKCδ, JNK, and P3 in DMED rats penile tissue obtained with LCG treatment, we also performed RT-qPCR and Western blot analysis. In comparison with the control group, the mRNA expression of CaSR, PLC, PKCδ, JNK, and P38 was significantly increased, yet significantly decreased, in the middle- or high-dose group, HTQG, and U73122 group (Figure 7B–F). While no obvious change was found in the low-dose LCG group (*P* > 0.05). Furthermore, the results of Western blot analysis suggesting that the protein expression of CaSR, PLC, PKCδ, JNK, and P38 were significantly elevated in the DMED group (Figure 7A–F). Simultaneously, the gray values of CaSR, PLC, PKCδ, JNK, and P38 expression were significantly diminished in the middle- or high-dose group, HTQG, and U73122 group but not in the low-dose LCG group, which are consistent with the result of mRNA expression in the present study.

## Discussion

In recent years, diabetic patients are also suffering from ED with increased susceptibility, affecting seriously male health and psychology. Although efforts have been made to investigate novel therapeutic avenues for DMED, including gene therapy, transplantation of stem cell, anti-PDF5 therapy and low energy shock wave therapy [28]. However, the strategy contributes to ameliorate DM as well as ED simultaneously is still under investigation and the mechanism of DMED is largely unknown. TCM believes that the etiology and pathogenesis of ED are associated with qi and blood block. Modern research shows that Chinese medicine can promote blood circulation and improve the blood supply to aid in the cavernous congestion [29]. The Leech is a blood-sucking annelid worms, which was first recorded in Shen Nong’s Herbal Classic. It has effects on promoting blood circulation, alleviating blood coagulation, activating meridians and relieving stasis by using alone or with other medicines with the dried whole body that used as a traditional TCM [30]. Chinese red-headed centipede, predatory terrestrial arthropods, belongs to the class Chilopoda and is characterized by a head and an externally segmented body with a pair of articulated legs for each segment that feeds with reptiles, bats, rats, and so on [31]. Centipede, an ancient antipyretic medicine used for centuries in traditional Chinese with the dried whole body that is processed using a special technique called PaoZhi, which is salted in flavor, warm in nature and attributives to the liver, spleen and lung meridians according to the Pharmacopoeia of the People’s Republic of China. It also can improve blood microcirculation and relieve blood stasis with calm the wind and activating collaterals, attacking toxin and regulating Qi [32].

In the present study, we attempted to demonstrate the therapeutic effect and the molecular mechanism associated with Leech and Centipede Granules in the penis tissues of DMED rats. The DMED rats generally underwent a higher fasting blood glucose, severe body weight and insulin loss, and glucagon increase. Initially, the present study revealed that compared with DMED rats, rats with LCG administration significantly improved the aforementioned serious symptoms. Additionally, the numbers of erection measurements were utilized for the evaluation of erectile function preliminarily. We found that LCG as a therapeutic agent for DMED can significantly aid in the erectile function restoration. Moreover, the results of our study revealed that impairment of the vascular endothelial of the penis was noted to be alleviated with the inhibition of the LOX-1, NOX, and EMP.

As is known, oxidative stress is well recognized as a major pathogenic factor in the pathogenesis of DMED, which characterized by overproduction of ROS [33] and induces endothelial cell injury with the reduction of NO [34]. Numerous reports have indicated that NO plays a crucial role in the induction and maintenance of normal vascular physiology, is released from noncholinergic nerves and endothelium [16,35], and has been clarified that its bioavailability is decreased in the penis of human DMED patients [10,36]. The erectile response and maintain erection relies on the production of NO via nNOS initiates and eNOS, respectively. Further, eNOS is the key enzyme for NO synthesis, which is expressed in the vascular endothelium widely. Moreover, NO plays an important role in ameliorating tissue fibrosis. In addition, Ferrini et al. found that iNOS inactivation caused exacerbated penile fibrosis in DM mice by increasing oxidative stress under hyperglycemic conditions [37]. Besides, ET-1, a potent vasoconstrictor, and proinflammatory peptide is the predominant isoform expressed in the vasculature, which released mostly by vascular endothelial cells and is another major regulator of vascular tone [38]. A previous report of Mao et al. has highlighted a correlation for the imbalance between ET-1 and NO systems in the pathogenesis of ED under cardiac hypertrophy [39]. Consistent with the reported previously, our current study showed that rats in DMED group elucidated by decreased NO and eNOS levels, and increased the potent vasoconstrictors, ET-1 content in penile tissues, as well as excessive concentration of early vascular fibrosis biomarkers such as VCAM-1, ICAM-1, and CD62P in the corpus cavernosum. In contrast, a significant increase of NO and eNOS, and markedly decrease of ET-1, VCAM-1, ICAM-1, and CD62P were observed in the LCG and U73122 treatment groups. In the present study, histological changes also observed by staining results reflected the protective effect of LCG on penile tissue endothelial cells. Taken together, these findings elucidated that the therapeutic impact of LCG on DMED in the diabetic penis may be due to improve vascular endothelial injury, the ability of vasodilation and decrease the penile fibrosis.

Evidence has been presented verifying that DM also has a tight correlation with the apoptosis of various cell types, including the cavernous smooth muscle cells [40], corpus cavernosal endothelial cell [10]. Apoptosis is a basic programmed cell death process to maintain the dynamic balance of the cell environment. The Bcl-2 family consists of the anti-apoptotic protein Bcl-2 and the pro-apoptotic protein Bax. Bcl-2 is a famous representative suppressor of apoptosis, while Bax can combine with Bcl-2 to form a heterodimer for antagonize the inhibitory effect of Bcl-2 on apoptosis and promote apoptosis [41]. On the other hand, Bax also activates caspase-3 with promoting the Ca^2+^ release and then causes cell apoptosis [42]. Caspase-3 is as a representative executor plays a pivotal role in cell apoptosis. Therefore, the Bax, Bcl-2, and caspase-3 levels are considered to be an indicator for evaluation of apoptosis. In our study, we also found that compared with that in the control group, the protein expression levels of BAX and caspase-3 were up-regulated and that Bcl-2 was down-regulated in the DMED group via immunohistochemical analysis, which agrees with previous studies [10,11]. Encouragingly, the protein level of Bcl-2 in the penile tissue of the LCG group significantly increased, and the protein expression of ax and the Caspase-3 decreased significantly in the penile tissue, indicating that LCG can effectively reduce the degree of apoptosis in the cavernous tissues of DMED rats.

It is established that the cytoplasmic free calcium concentration associated with various functions of vascular endothelium, and has significant roles in regulating vascular tone and the generation of NO [43]. The CaSR, a PLC sensitive G protein-coupled receptor, can increase the release of [Ca^2+^]i from the endoplasmic reticulum via the inositol triphosphate receptor (IP3R). Notably, Liu et al. demonstrated that CaSR activated PKCδ to induce cardiomyocyte apoptosis during ischemia/reperfusion (I/R) injury [44]. However, the associations between CaSR, PLC, PKCδ, and DMED are not clear. Our qPCR and Western blot results suggest that LCG might attain its effects via inhibition of the CaSR/PLC/PKC pathway, as well as the mRNA and protein expression of JNK, P38, and BAX. Consistent with the results of LCG, the present study showed the U73122, a PLC inhibitor helps to eliminate the deterioration of ED in the present study. Thus, we concluded that LCG may enhance the NO level to ameliorate erectile function via decreasing cell apoptosis by inactivation of the CaSR/PLC/PKC pathway. There are many defects of our study, such as the only Type 2 diabetic ED rats were investigated, and the Ca^2+^ concentration should be measured in penile tissue. Besides, further the gain-of-function and loss-of-function cell assays are needed. Therefore, we will also reconfirm the cellular effects and accurate mechanism of LCG in our future studies.

In the present study, we demonstrated that the couplet medicines of Leech and Centipede Granules ameliorate erectile function in DMED rats, and the inactivation of CaSR/PLC/PKC signaling pathway by the couplet medicines of Leech and Centipede Granules were responsible for the reduction of endothelial cells apoptotic death.

## Abbreviations

AGE: advanced glycation end
APC: allophycocyanin
APO: apomorphine
CD62P: selectin P
cGMP: cyclic guanosine monophosphate
DAB: diaminobenzidine
DAG: diacylglycerol
DM: diabetes mellitus
DMED: diabetes mellitus-induced erectile dysfunction
ED: erectile dysfunction
EMPs: endothelial microparticles
eNOS: endothelial NOS
GPCR: G-protein coupled receptor
HTQGs: Huoxue Tongluo Qiwei soup granules
I/R: ischemia/reperfusion
ICAM-1: intracellular adhesion molecule-1
IHC: immunochemistry
IP3R: inositol triphosphate receptor
LCG: Leech and Centipede Granules
LOX-1: lectin-like oxidized low-density lipoprotein receptor-1
MAPK: mitogen-activated protein kinase
NO: nitric oxide
NOX: NADPH-oxidases
PDE5: phosphodiesterase type 5
PE: Phenylethylamine
PKC: Protein kinase C
PLC: phospholipase C
PPP: prepare platelet-poor plasma
PRP: platelet-rich plasma
ROS: reactive oxygen species
SD: Sprague-Dawley
SDS-PAGE: sodium dodecyl sulfate/polyacrylamide gel electrophoresis
TCM: traditional Chinese medicine
VCAM-1: vascular cell adhesion molecule-1
