# Correction: Couplet medicines of leech and centipede granules improves erectile dysfunction via inactivation of the CaSR/PLC/PKC signaling in streptozotocin-induced diabetic rats

**DOI:** 10.1042/BSR-2019-3845_COR

**Published:** 2023-01-19

**Keywords:** apoptosis, CaSR/PLC/PKC signaling pathway, diabetes mellitus, endothelial cells, erectile dysfunction

The authors of the original article “Couplet Medicines of Leech and Centipede Granules Improves Erectile Dysfunction via Inactivation of the CaSR/PLC/PKC Signaling in Streptozotocin-induced Diabetic Rats” (Biosci Rep. 2020 40(2): BSR20193845. https://doi.org/10.1042/BSR20193845) would like to correct Figure 5, following a notification from a reader identifying a duplication.

**Figure 5:**
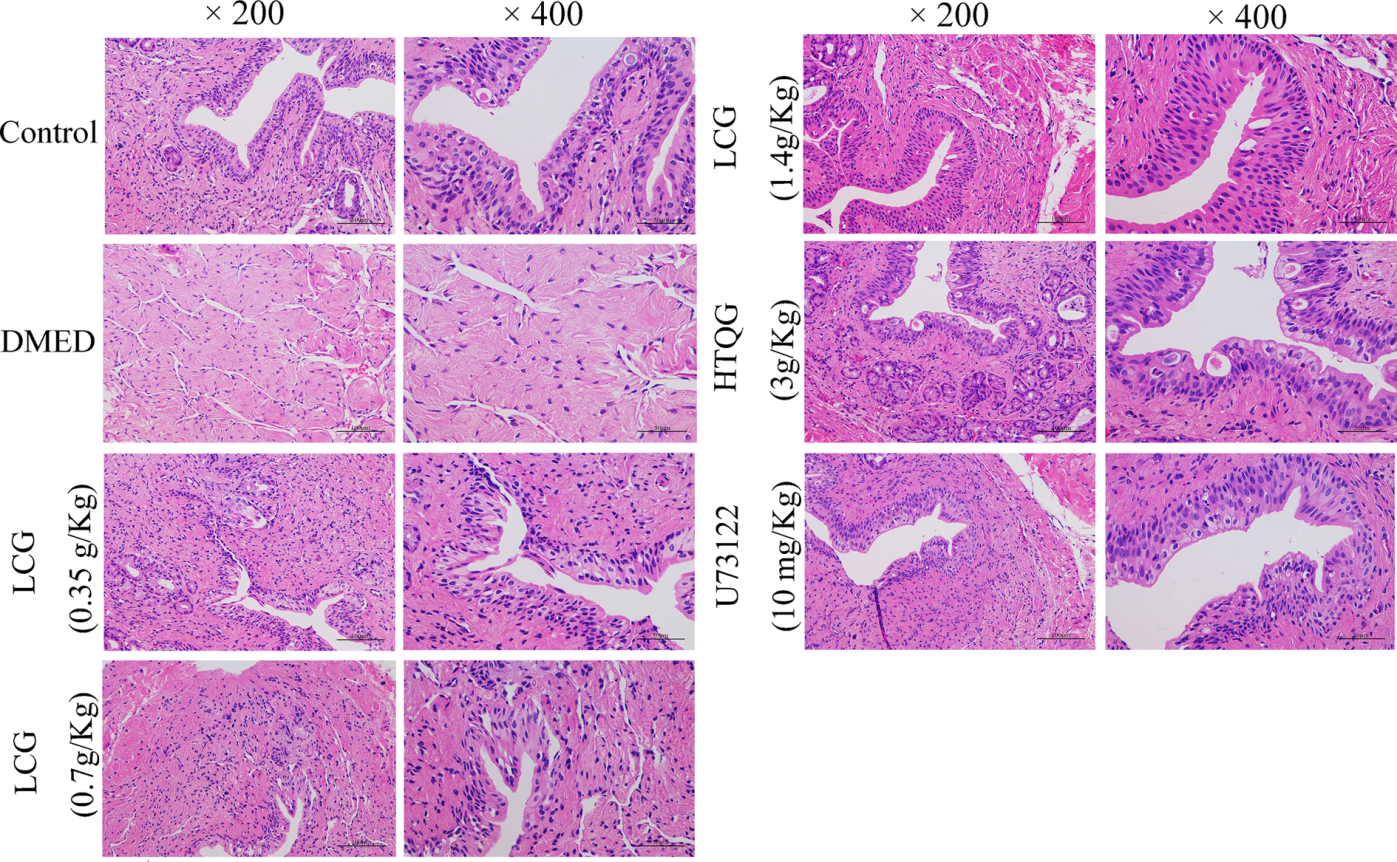
Effect of LCG on the pathological progression of DMED Pathological changes of cavernous bodies of rats treated with LCG at the dosage of 0.35 g/kg, 0.7 g/kg, and 1.4 g/kg were observed by hematoxylin and eosin staining (200× and 400×)

The authors state that the images used in the original Figure 5, when composing the HE images, were incorrectly selected. The authors have provided the Editorial Office with the raw data for the original and corrected Figure. The requested correction has been assessed and agreed by the Editorial Board. The authors declare that these corrections do not change the results or conclusions of their paper. The corrected version of Figure 5 is presented here.

